# Combined Effects of Lung Volume Recruitment Training and Mechanical Insufflation–Exsufflation in a Patient With Advanced Amyotrophic Lateral Sclerosis Receiving Long-Term Mechanical Ventilation: A Case Report

**DOI:** 10.7759/cureus.83823

**Published:** 2025-05-09

**Authors:** Shino Sue, Shinobu Yamazaki, Keita Sue, Tomomi Kinoshita, Kunihiro Yoshida

**Affiliations:** 1 Department of Physical Therapy, JA Nagano Kouseiren Kakeyu-Misayama Rehabilitation Center Kakeyu Hospital, Ueda, JPN; 2 Department of Fundamental Physical Therapy, School of Medicine, Shinshu University, Matsumoto, JPN; 3 Department of Neurology, JA Nagano Kouseiren Kakeyu-Misayama Rehabilitation Center Kakeyu Hospital, Ueda, JPN

**Keywords:** amytrophic lateral sclerosis, invasive mechanical ventilation, lung compliance, lung volume recruitment, mechanical insufflation-exsufflation, neurogenerative diseases, pulmonary rehabilitation and medicine, respiratory physiotherpay, respiratory support, tracheostomy ventilation and amyotrophic lateral sclerosis

## Abstract

Amyotrophic lateral sclerosis (ALS) degenerates both upper and lower motor neurons. Most patients with ALS require respiratory support due to deterioration of their respiratory muscles. Mechanical insufflation-exsufflation (MI-E) is one option that can help patients with weak cough strength to clear the airway, and it may potentially increase survival time. Another option is lung volume recruitment training (LVRT), a technique commonly used to maintain lung and chest wall flexibility. However, it requires specific equipment, such as one-way valves, to be applied to patients with ALS who undergo invasive mechanical ventilation with tracheostomy. Only limited studies have indicated the effectiveness of LVRT for patients with ALS. Moreover, no study is currently available on the effect of combining LVRT with MI-E. As the disease progresses, treatment options become increasingly limited, making it crucial to explore new therapeutic approaches for patients at the advanced stage. Here, we examined the effects of a combination of LVRT and MI-E in a 74-year-old female patient with ALS who had survived under invasive mechanical ventilation for nine years. We measured tidal volume (TV) and dynamic lung compliance (Cdyn) as respiratory parameters three months before and after the initiation of the combined therapy. Following the intervention, TV improved from 750.15 L/min (standard deviation (SD) ± 34.60) to 859.14 L/min (SD ± 75.63), and Cdyn increased from 24.18 cmH₂O (SD ± 2.84) to 26.54 cmH₂O (SD ± 2.92). These results suggest that MI-E combined with LVRT may improve lung compliance even in patients with ALS receiving long-term invasive mechanical ventilation.

## Introduction

Amyotrophic lateral sclerosis (ALS) is a fatal neurodegenerative disease with no radical cure available [1]. Muscle wasting and weakness gradually advance as a result of degeneration of the upper and lower motor neurons in patients with ALS [1]. Respiratory failure is a serious complication due to the loss of bulbar, cervical, and thoracic motor neurons [2]. Physical therapy (PT) is a common intervention to maintain and/or improve respiratory functions in ALS [3], and it is effective even in the advanced stage [4]. PT for respiratory functions includes inspiratory muscle training, lung volume recruitment training (LVRT), and manually assisted cough [3]. However, the effectiveness of PT by itself gradually decreases as the disease progresses. When respiratory muscles deteriorate [3], alternative techniques are needed to maintain respiratory function in addition to non-invasive or invasive mechanical ventilation [5]. In particular, mechanical insufflation-exsufflation (MI-E), also known as "cough assist," is a technique used to enhance cough peak flow, clear airway mucus [6], and reduce the risk of atelectasis [7]. The device supports patients with impaired cough ability by promoting the clearance of respiratory secretions and can be utilized both with and without mechanical ventilation [8]. The combination of mechanical ventilation and MI-E potentially increases survival time [9]. It may also benefit patients in the late stage of ALS by relieving their self-perceived cough deficiency [10].

LVRT is a commonly used stacked-breath-assisted inflation technique to maintain lung and chest wall flexibility and to slow the decline of respiratory function in patients with neurodegenerative diseases [11]. LVRT has been shown to improve maximum insufflation capacity (MIC) in adults with neuromuscular disease compared to conventional breathing exercises [12]. However, this technique requires patients to stack their breath, and most patients under invasive mechanical ventilation with tracheostomy have difficulty performing breath stacking. For patients with such difficulties, LVRT using a manual resuscitation bag equipped with specific valves [13,14] was developed as an alternative. Previous studies have determined that continuous LVRT with those valves could improve lung capacity in patients with tracheostomized neuromuscular diseases, including ALS [13,15].

While LVRT is a crucial method for improving respiratory function [11], its application in patients with tracheostomy is challenging without specialized devices [13-15]. Additionally, the efficacy of combined MI-E and LVRT therapy in ALS patients undergoing long-term invasive mechanical ventilation remains largely unknown. The purpose of this case report was to determine whether combination therapy could effectively improve respiratory functions in the advanced stage of ALS.

This article was previously presented as a meeting abstract at the 22nd Congress of the Japanese Society of Neurological Physical Therapy on September 28, 2024.

## Case presentation

Patient history and clinical presentation

The patient was a 74-year-old elderly woman who first noticed difficulty in speaking at age 59. She experienced difficulty in swallowing soon after. At the neurology clinic, she was found to have tongue atrophy and fasciculation. She was referred to the university hospital and diagnosed with the bulbar type of ALS at age 60. Riluzole (100 mg/day) was initiated. As her swallowing difficulty gradually worsened, percutaneous endoscopic gastrostomy (PEG) was performed for nutritional support at age 61. At that time, she remained independent in writing, using a mobile phone, and walking, despite severe difficulty in speaking and swallowing.

As the disease progressed, she experienced episodes where phlegm nearly obstructed her throat, which led to a tracheostomy at age 62. She was subsequently transferred to our hospital, where she remained hospitalized to undergo care and treatment, including physical therapy. For a while afterward, she survived on spontaneous breathing; however, during this period, her respiratory function gradually declined. She was intermittently supported by mechanical ventilation, particularly at night when she experienced respiratory discomfort. By age 65, she required continuous mechanical ventilation.

At present, she was totally bedridden and had completely lost the function of her hands and feet. However, she was alert and able to respond to closed-ended questions by contracting her facial muscles or moving her eyes. Her body mass index (BMI) was 18.84 kg/m². Deep tendon reflexes were absent. Pathological reflexes, such as Babinski and Hoffmann's reflex, were negative. The ALS Functional Rating Scale-Revised (ALSFRS-R) score was 0 out of 46. Activities of daily living were assessed using the Functional Independence Measure (FIM), with a total score of 19 out of 126 (motor: 13; cognition: 6).

Spontaneous breathing was not observed, and she was entirely dependent on a mechanical ventilation system (LTV2200, Vyaire Medical, Inc., US) via tracheostomy. Volume control mode, synchronized intermittent mandatory ventilation (SIMV) with positive end-expiratory pressure (PEEP), was applied.

Physical therapy, including MI-E

PT had been conducted for approximately 40 minutes per session on weekdays since her hospitalization. It included MI-E using Cough Assist (E70®, Philips Respironics, Murrysville, Pennsylvania) for clearing the airway and maintaining respiratory functions, in addition to mobilization to prevent the contraction of whole-body joints. MI-E was first introduced to clear increased airway mucus at age 70. The settings were adjusted to enhance mucus clearance at age 71. Since then, MI-E has been performed in auto mode without the CoughTrack algorithm or oscillation. It was conducted five times × two sets with expiratory pressure +25 cmH₂O and inspiratory pressure -25 cmH₂O at 30° head-up position in daily PT sessions. The inspiratory and expiratory times (TI and TE) were set at 1.5 seconds, and the inspiratory and expiratory pause times were also set at 1.5 seconds. Vital signs such as oxygen saturation levels, heart rate, and breathing rate were carefully monitored during MI-E. The first set was primarily aimed at mucus clearance, while the second set focused on maintaining respiratory function, particularly dynamic lung compliance (Cdyn).

**Figure 1:**
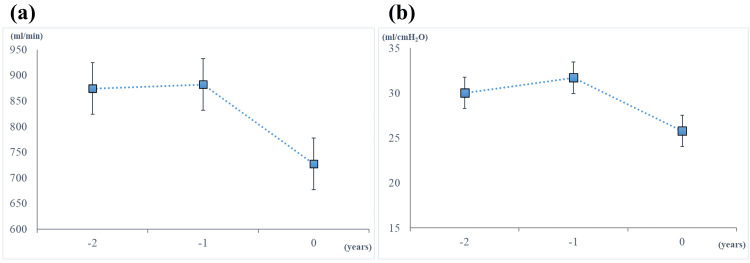
Serial change of monitored respiratory parameters. (a) Tidal volume. (b) Dynamic lung compliance. The block symbol represents the mean value measured over seven consecutive days in the same month across three consecutive years. The error bar represents the standard deviation. Zero on the X-axis indicates the year when LVRT was added to MI-E. LVRT was initiated just after the measurement at zero. Negative one and negative two on the X-axis represent the evaluation points one year and two years before the introduction of LVRT, respectively. LVRT: lung volume recruitment training, MI-E: mechanical insufflation-exsufflation.

To evaluate the efficacy of MI-E, we monitored the parameters, including TV (L/min), peak inspiratory pressure (PIP) (cmH₂O), and PEEP (cmH₂O). TV was obtained from MI-E, while PIP and PEEP were obtained from the mechanical ventilator. Cdyn (mL/cmH₂O) was calculated by TV/(PIP - PEEP). The serial changes in TV and Cdyn for the past two years just before this study are illustrated in Figure 1. These respiratory parameters were stable for the first year, but thereafter declined, despite continued intervention with MI-E. This prompted us to newly introduce LVRT in addition to MI-E.

Study protocol: methods for combined therapy of LVRT and MI-E

LVRT was combined with MI-E in daily practice to improve her respiratory function, especially in Cdyn. In LVRT, the LIC trainer® (Carter Technologies Inc., Japan), which can measure LIC and attain LVRT for patients with tracheostomy [16], was used. The details of the function and usage of the LIC trainer® are shown elsewhere [16]. In brief, this equipment is composed of a one-way valve, a safety valve, and an expiration release valve. A resuscitation bag attached to a one-way valve sends air to patients through a bag valve mask without adverse tide. The safety valve has four grade adjustments ranging from minimum first grade (20 to 30 cmH₂O) to maximum fourth grade (50 to 60 cmH₂O) to prevent lung injury by positive pressure. Patients who can use their hands or operators can control their breath using the expiration release valve.

In PT sessions, LVRT was conducted after the first MI-E session to clear mucus. The physical therapists delivered pressure to the patient in the same 30° head-up position using a resuscitation bag attached to the LIC trainer®, adjusted at the first grade, five times across three sets in a day. Each time, the physical therapists delivered pressure, held the air for three seconds as an air stack, and then released the stacked air. After LVRT, MI-E was conducted again to improve respiratory function and measure respiratory parameters.

We observed respiratory parameters three months before and after the initiation of LVRT with MI-E. The monitored parameters included TV (L/min), PIP (cmH₂O), and PEEP (cmH₂O). TV was obtained from MI-E, while PIP and PEEP were obtained from the mechanical ventilator. Cdyn (mL/cmH₂O) was calculated by TV / (PIP - PEEP). We recorded the fifth TV value in the second session. All parameters were expressed as mean ± SD. To confirm the degree of changes before (M^a^) and after (M^b^) the initiation of LVRT, effect sizes (standard mean difference (SMD) (d)) were calculated using the following:

$d = \frac{M^{b}-M^{a}}{SD_{pool}}$

In addition, trends in TV and Cdyn were visually assessed by plotting daily values on a graph, with celeration lines used to illustrate changes before and after the initiation of LVRT.

Ethical approval

The study protocol was approved by the Medical Ethics Committee of Kakeyu Hospital (Approval number: 2023024). The written informed consent was obtained from the patient’s family because the patient was incapable of writing.

Results

**Figure 2:**
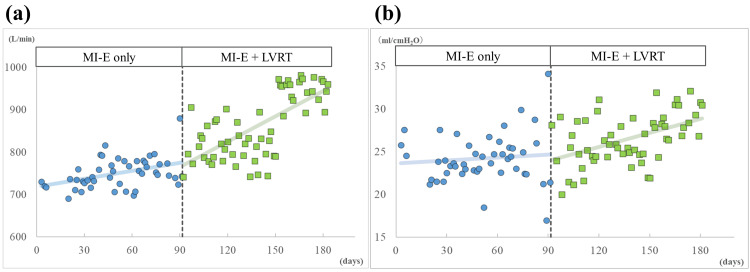
Changes in monitored respiratory parameters three months before and after the initiation of LVRT. (a) Tidal volume, (b) Dynamic lung compliance. Data points before and after the initiation of LVRT are represented by blue circle and green block symbols, respectively. Celeration lines before and after the initiation of LVRT are represented by blue and green colors, respectively. The periods of MI-E and combined therapy (MI-E and LVRT) could be divided by the vertical dashed line. LVRT: lung volume recruitment training, MI-E: mechanical insufflation-exsufflation

The mean TV values three months before and after LVRT initiation were 750.15 L/min (SD ± 34.60) and 859.14 L/min (SD ± 75.63), respectively. The mean Cdyn values before and after LVRT were 24.18 cmH₂O (SD ± 2.84) and 26.54 cmH₂O (SD ± 2.92), respectively. Improvements in TV and Cdyn were confirmed after combining LVRT with MI-E. The effect sizes (d) were 1.80 in TV and 0.79 in Cdyn. The values of TV and Cdyn three months before and after the initiation of LVRT are presented in Figure 2, showing upward acceleration lines in both parameters following the introduction of LVRT. In response to the positive results described above, we are continuing this combination therapy in PT sessions.

## Discussion

We reported a case of a patient with ALS who had been under invasive mechanical ventilation for nine years and examined the effects of combined MI-E and LVRT therapy on respiratory function. While LVRT is a crucial method for improving respiratory function [11], its application in patients with tracheostomy is challenging without specialized devices [13-15]. Additionally, the efficacy of combined MI-E and LVRT therapy in ALS patients undergoing long-term invasive mechanical ventilation remains largely unknown. TV and Cdyn in this patient improved after initiating the combined therapy, and this indicated that the combination may improve respiratory functions in patients with advanced ALS.

MI-E was introduced to the patient at age 70 to clear excessive airway mucus and maintain respiratory function. Looking at the past two years just before this study (Figure 1), her TV and Cdyn values were maintained for the first year; however, they showed a marked decrease in the following year. These declines in respiratory parameters may be attributed to reduced lung compliance associated with long-term ventilation, often caused by chest wall abnormalities [17]. Aging may also have contributed to this deterioration [18]. The time course of her respiratory parameters indicates that despite the use of MI-E, a well-established technique for preserving respiratory function in patients with neurodegenerative diseases [8], lung compliance may decline inevitably under prolonged mechanical ventilation. Nevertheless, our findings revealed improvements in both TV and Cdyn following the introduction of LVRT. The calculated SMD (d) values indicated a large effect size for both parameters. Clear upward trends in both TV and Cdyn over time (Figure 2) suggest that the combination of MI-E and LVRT may enhance lung compliance, even in patients with advanced ALS under long-term invasive mechanical ventilation.

This case suggests that LVRT may enhance the effectiveness of MI-E, offering additional benefits for respiratory function. Given that treatment options become increasingly limited as ALS progresses, this case provides new insights into potential therapeutic approaches for patients in the advanced stages of the disease.

We have presented a favorable result of the combination of MI-E and LVRT, but several concerns remain. First, it is not clear whether the combination therapy or LVRT alone was effective in improving respiratory function in this patient. MI-E could not be excluded from daily practice to confirm the effects of LVRT alone because the patient needed to clear airway mucus daily. Second, the optimal timing for introducing LVRT in combination therapy remains to be clarified. Third, the effect of pressure settings in the LIC Trainer® on LVRT remains unclear. Finally, it is unknown how long the observed effects will last. Further studies are necessary to determine better methods for combination therapy targeting respiratory parameters in patients with advanced ALS.

## Conclusions

We confirmed the effects of combined LVRT with MI-E on respiratory function in a patient with advanced ALS who had undergone long-term invasive mechanical ventilation. The results showed that both TV and Cdyn improved after the initiation of the combined therapy, with large effect sizes, providing insight into respiratory support for patients with advanced ALS.
